# Periodontitis and osteoporosis: a two-sample Mendelian randomization analysis

**DOI:** 10.1590/1414-431X2024e12951

**Published:** 2024-03-18

**Authors:** Jiale Wu, Lihui Yao, Yuchen Liu, ShuaiShuai Zhang, Kan Wang

**Affiliations:** 1 Department of Oral and Maxillofacial Surgery, Peking University School of Medicine, Hospital of Stomatology, Beijing, China; 2 Department of Stomatology, The First Affiliated Hospital of Zhengzhou University, Zhengzhou, Henan, China

**Keywords:** Periodontitis, Osteoporosis, Mendelian randomization, Genetics, Causal association

## Abstract

The incidences of periodontitis and osteoporosis are rising worldwide. Observational studies have shown that periodontitis is associated with increased risk of osteoporosis. We performed a Mendelian randomization (MR) study to genetically investigate the causality of periodontitis on osteoporosis. We explored the causal effect of periodontitis on osteoporosis by MR analysis. A total of 9 single nucleotide polymorphisms (SNP) were related to periodontitis. The primary approach in this MR analysis was the inverse variance-weighted (IVW) method. Simple median, weighted median, and penalized weighted median were used to analyze sensitivity. The fixed-effect IVW model and random-effect IVW model showed no significant causal effect of genetically predicted periodontitis on the risk of osteoporosis (OR=1.032; 95%CI: 0.923-1.153; P=0.574; OR=1.032; 95%CI: 0.920-1.158; P=0.588, respectively). Similar results were observed in simple mode (OR=1.031; 95%CI: 0.780-1.361, P=0.835), weighted mode (OR=1.120; 95%CI: 0.944-1.328, P=0.229), simple median (OR=1.003; 95%CI: 0.839-1.197, P=0.977), weighted median (OR=1.078; 95%CI: 0.921-1.262, P=0.346), penalized weight median (OR 1.078; 95%CI: 0.919-1.264, P=0.351), and MR-Egger method (OR=1.360; 95%CI: 0.998-1.853, P=0.092). There was no heterogeneity in the IVW and MR-Egger analyses (Q=7.454, P=0.489 and Q=3.901, P=0.791, respectively). MR-Egger regression revealed no evidence of a pleiotropic influence through genetic variants (intercept: -0.004; P=0.101). The leave-one-out sensitivity analysis indicated no driven influence of any individual SNP on the association between periodontitis and osteoporosis. The Mendelian randomization analysis did not show a significant detrimental effect of periodontitis on the risk of osteoporosis.

## Introduction

Periodontitis is a common illness characterized by an inflammatory condition of the dental supporting tissues induced by several factors (1,2). Globally, 1.1 billion cases of severe periodontitis were reported in 2019 (95%CI: 0.8-1.4 billion). The worldwide incidence of severe periodontitis increased by 8.44% (6.62 to 10.59%) from 1990 to 2019. Severe periodontitis is more common in less developed nations. From 1990 to 2019 (3), 67.9% of the increase in severe periodontitis was attributed to global population growth. Older adults are more likely to develop periodontitis. Periodontitis is significantly associated with various chronic illnesses, like cardiovascular and cerebrovascular conditions, tumors, chronic kidney disease, and depression (4-7).

Osteoporosis is a worldwide health concern. Fractures due to osteoporosis contribute significantly to morbidity and mortality worldwide. Although postmenopausal osteoporosis is the most common form, secondary osteoporosis affects up to 30% of postmenopausal women, more than 50% of premenopausal women, and 50 to 80% of men. As addressing the underlying disease is frequently the first step in treating such patients, it is crucial to rule out secondary causes (8). Several studies (9-11) have found that chronic periodontitis has a systemic effect, increasing the likelihood of developing osteoporosis. However, these studies were observational. Genetic evidence linking periodontitis and a higher risk of osteoporosis has not been adequately studied. As osteoporosis and periodontitis are complex illnesses with shared pathways, it is difficult to determine how they are related (12). The published observational causal relationships between osteoporosis and periodontitis are subject to bias.

Mendelian randomization (MR) has received much attention to protect against some major limitations in traditional observational research (13). In the two-sample MR, germline genetic variants are viewed as instrumental variables (IV) to investigate the causality between exposure and outcome phenotype. This method avoids specific problems in observational studies and applies publicly accessible information from significant genome-wide association studies (GWASs) for exposure and outcome. GWAS datasets include regression estimates of the exposure or outcome of the genetic variant. In this study, we conducted a two-sample MR study to investigate the causal relationship between periodontitis and osteoporosis.

## Material and Methods

### Methodology and data sources

**Figure 1:**
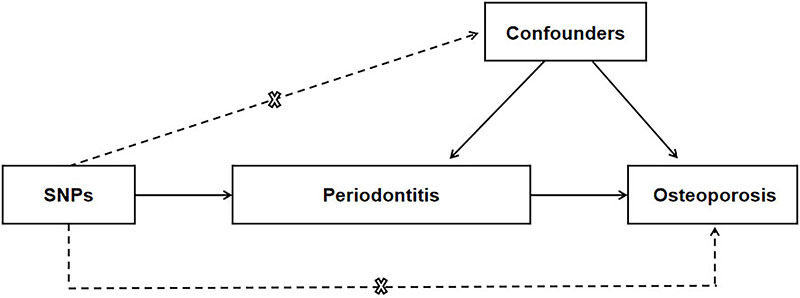
Mendelian randomization model of periodontitis and risk of osteoporosis. The design is under the assumption that the genetic variants are associated with periodontitis, but not with confounders, and the genetic variants influence osteoporosis only through periodontitis. SNPs: single nucleotide polymorphisms.

This MR analysis assessed the effect of periodontitis on osteoporosis (Figure 1). We gathered instrumental variables of periodontitis from the latest GWAS meta-analysis regarding Gene-Lifestyle Interaction in the Dental Endpoints (GLIDE) Consortium. To this day, the sample capacity was the largest, containing 17,353 clinically identified instances and 28,284 controls. The outcome of this research was osteoporosis. The osteoporosis summary values were derived from the largest GWAS recently released (ID: bbj-a-137; https://gwas.mrcieu.ac.uk/datasets/bbj-a-137/). We used data summarized in previous studies, which have permission from relevant institutional review boards. Therefore, no additional ethical approval was necessary.

### Selection of SNPs

The single nucleotide polymorphisms (SNPs) for periodontitis were derived from newly published GWAS conducted by Gene-Lifestyle Interaction in Dental Endpoints (GLIDE) Consortium. As preliminary candidate SNPs, autosomal biallelic SNPs with P-values <5×10^−5^ and minor frequencies >1% were chosen. In addition, we validated the independence of the selected genetic variations by grouping SNPs with linkage disequilibrium (R^2^ of 0.001 at a 10,000 kb frame). In the end, we identified 18 independent SNPs associated with periodontitis. Osteoporosis (ID: bbj-a-137; https://gwas.mrcieu.ac.uk/datasets/bbj-a-137) came from a recent GWAS dataset. This GWAS identified 8,885,805 SNPs to assess 212,453 individuals (7788 cases and 204,665 controls) of East Asian ancestry with systemic lupus erythematosus according to the disease characteristics database released by Biobank Japan. Based on the MR hypothesis, we extensively reviewed the literature to select osteoporosis risk factors (14-16). After searching these 18 SNPs in the web-based PhenoScanner V2 (http://www.phenoscanner.medschl.cam.ac.uk/) (17), none were associated with osteoporosis or risk factors for osteoporosis and 9 SNPs were not found in outcome data sets (ID: bbj-a-137; https://gwas.mrcieu.ac.uk/datasets/bbj-a-137). Thus, we used the remaining 9 SNPs as the instrument in the MR analysis. Table 1 provides all SNPs information used in this study.

**Table 1:** Detailed information of instrumental variables utilized in the Mendelian randomization (MR) analysis of periodontitis (PD) on osteoporosis.

SNP	Chr	Pos	EA/OA	EAF	Association with PD				Association with osteoporosis		
					β	SE	P-value	F-statistic	β	SE	P-value
rs10757466	9	24000258	T/C	0.14	-0.117	0.026	9.26E-06	19.704	-0.0064	0.019	0.739
rs13005050	2	52705571	T/C	0.88	-0.143	0.031	3.76E-06	21.332	0.0263	0.023	0.258
rs186040223	7	64014696	A/C	0.85	-0.158	0.035	5.66E-06	20.570	-0.0164	0.030	0.587
rs1901299	18	22442179	A/C	0.07	-0.090	0.020	8.63E-06	19.773	0.0069	0.021	0.739
rs28546695	18	49760987	A/G	0.38	0.083	0.019	8.21E-06	19.808	-0.0268	0.021	0.203
rs2976950	8	8249082	A/G	0.60	0.096	0.020	7.99E-07	24.382	-0.0196	0.025	0.443
rs4640758	5	7613236	A/G	0.36	-0.085	0.019	7.28E-06	20.120	-0.0002	0.018	0.990
rs4956201	4	109527782	A/C	0.06	-0.241	0.047	3.89E-07	25.768	-0.0402	0.023	0.081
rs6816769	4	122216017	T/C	0.08	-0.135	0.029	4.57E-06	21.020	-0.0411	0.049	0.404

SNP: single nucleotide polymorphisms; Chr: chromosome; Pos: position; EA/OA: effect allele/other allele; EAF: effect allele frequency.

### Analytical statistics

This study used 8 MR analysis tools. They are inverse-variance weighted (fixed-effect and random-effect), simple mode, simple median, weighted mode, weighted median, penalized weighted median, and MR Egger. Inverse-variance weighted (IVW) was the principal approach to explore the results since it offers a reliable causal estimate despite heterogeneity. As whole instrumental parameters should match the MR hypotheses in the IVW approach, we conducted sensitivity analyses using a weighted median estimator and MR-Egger. The weighted median estimator can produce reliable causal estimates when the validity of the instrumental variables exceeds 50%. The MR-Egger estimate is unbiased if the genetic instrument is independent of pleiotropic effects. Additionally, we evaluated the heterogeneity and pleiotropy of individual SNPs using IVW techniques with Cochran's Q statistics and MR Egger intercept. As long as the intercept was not significantly distinct from 0 (P>0.05), pleiotropy impacts were deemed absent. Cochrane's Q value estimates the heterogeneity. If the P-value was less than 0.05, the primary outcome was the IVW approach with a multiplicative random-effects model; otherwise, we applied the IVW method with a fixed-effects model. This research also used MR-Egger regression, which can detect and modify pleiotropy, estimate a causal impact, and evaluate the influence of directed horizontal pleiotropy on results. Furthermore, a leave-one-out analysis validated the robustness of the MR analysis outcomes in the presence of any outlier SNP. According to the prior study, the causal association was declared significant if the following three requirements were met: 1) The IVW P-value is less than 0.05; 2) The IVW, weighted median, and MR-Egger estimate all point in the same general direction; 3) A P-value of more than 0.05 was obtained for the MR-Egger intercept test. The “TwoSampleMR” package was used to conduct all statistical analyses in R version 3.4.1 (R Foundation for Statistical Computing, Austria). A two-tailed P-value of 0.05 was deemed statistically significant.

## Results

### Effect of periodontitis on osteoporosis

**Figure 2:**
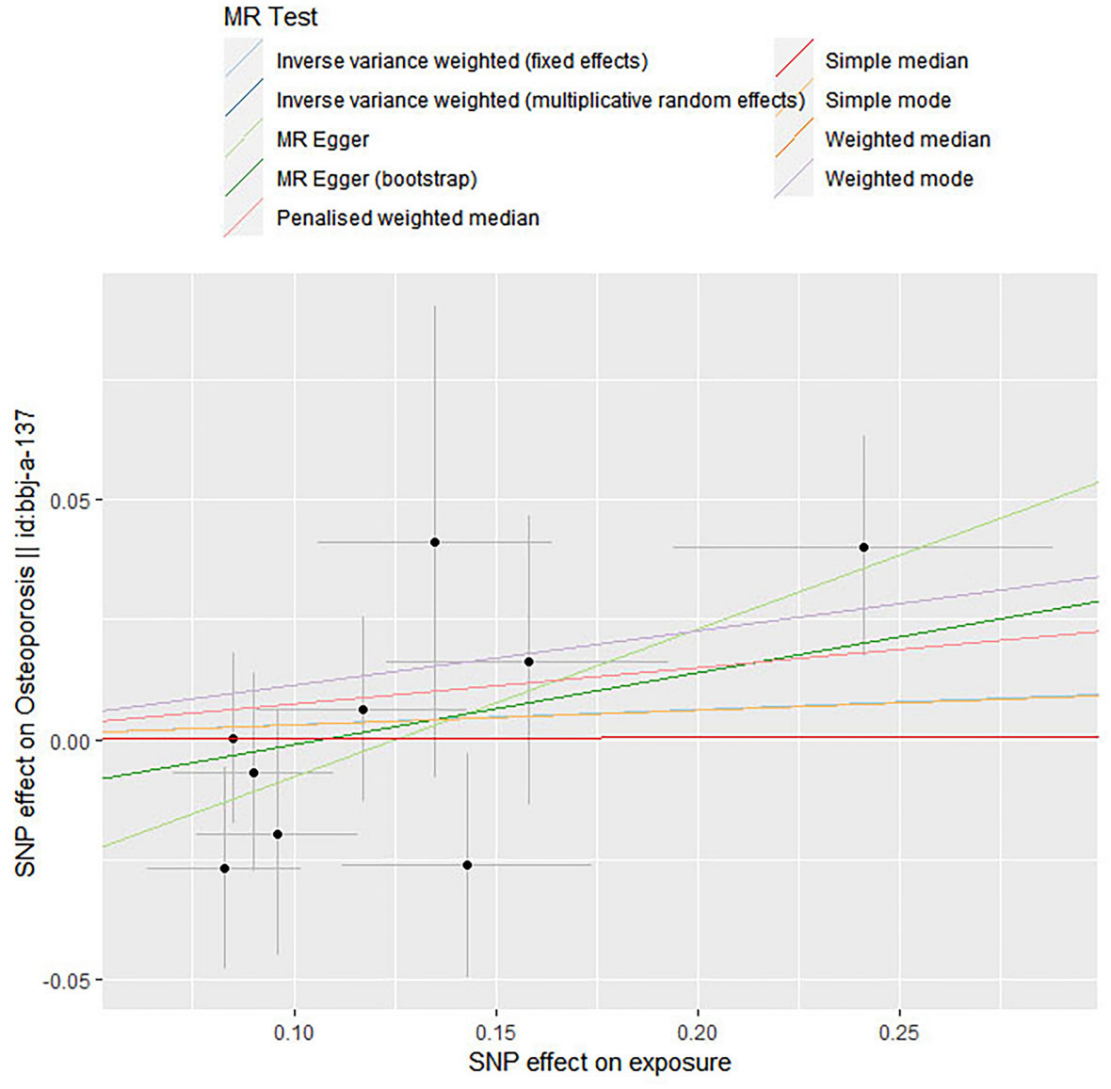
Scatter plot of causal effect of periodontitis on osteoporosis. The slopes of the straight lines indicate the magnitude of the causal association. SNP: single nucleotide polymorphism.

**Figure 3:**
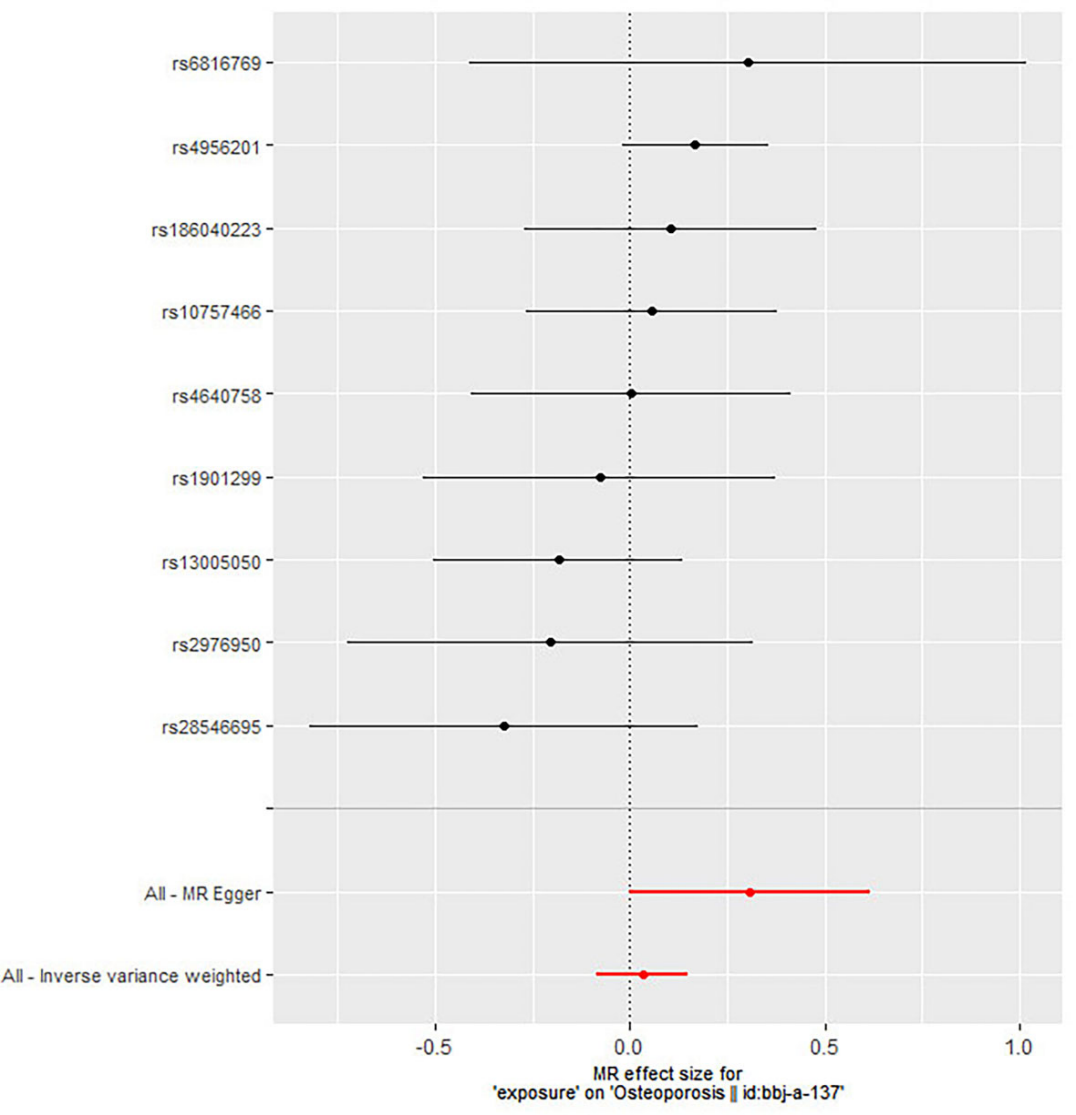
Fixed-effect inverse variance-weighted analysis of the causal association of periodontitis with osteoporosis. The black dots and bars indicate the causal estimate and 95%CI using each single nucleotide polymorphism (SNP). The red dots and bars indicate the overall estimate and 95%CI meta-analyzed by Mendelian randomization (MR)-Egger and fixed-effect inverse variance weighted method.

**Figure 4:**
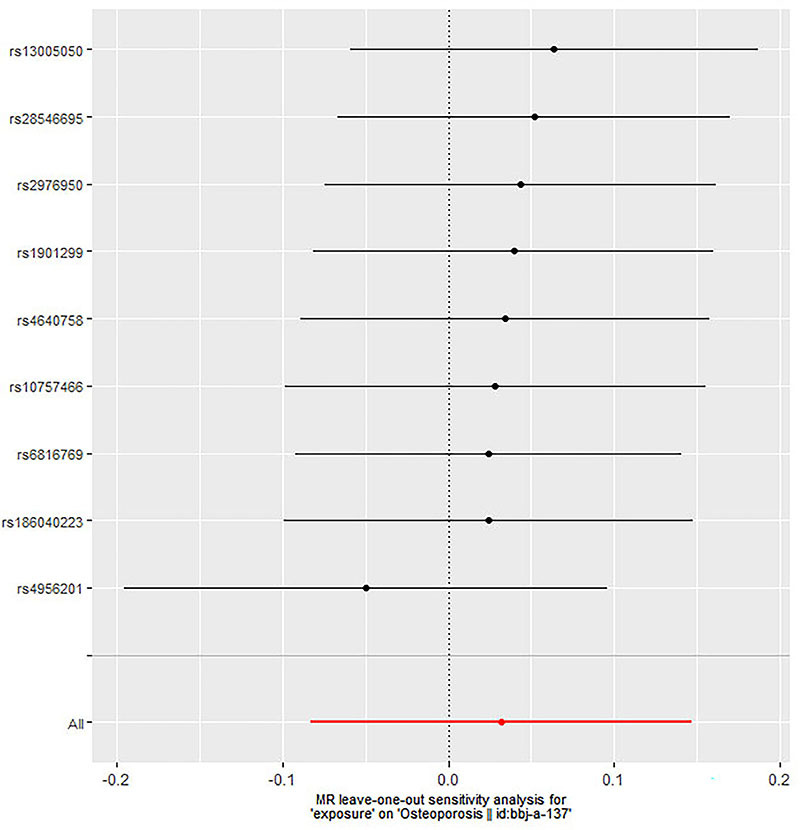
Mendelian randomization (MR) leave-one-out sensitivity analysis for a link between periodontitis and osteoporosis. Circles indicate MR estimates for periodontitis on osteoporosis using inverse-variance weighted fixed-effect method if each single nucleotide polymorphism (SNP) was omitted in turn.

Table 1 comprehensively describes the total genetic datasets employed in this research. Description of data sources and assessment of the instrumental variables’ strength are shown in Supplementary Table S1. Additional MR analysis confirmed the reliability of IVW analysis results, including simple mode, simple median, weighted mode, weighted median, penalized weighted median, and MR Egger (Figure 2). Each SNPs' pleiotropy and heterogeneity were assessed using IVW techniques, Cochran's Q statistics, and MR Egger intercept. In this MR, we identified 9 instrument variables. The fixed-effect and random-effect inverse-variance weighted models revealed no link between periodontitis and increased risk of osteoporosis (OR=1.032; 95%CI: 0.923-1.153; P=0.574; OR=1.032; 95%CI: 0.920-1.158; P=0.588, respectively). Similar results were observed in simple mode (OR=1.031; 95%CI: 0.780-1.361, P=0.835), weighted mode (OR=1.120; 95%CI: 0.944-1.328, P=0.229), simple median (OR=1.003; 95%CI: 0.839-1.197, P=0.977), weighted median (OR=1.078; 95%CI: 0.921-1.262, P=0.346), penalized weighted median (OR=1.078; 95%CI: 0.919-1.264, P=0.351), and MR-Egger method (OR=1.360; 95%CI: 0.998-1.853, P=0.092) (Figure 3 and Table 2). There was no heterogeneity in the IVW and MR-Egger analyses (Q=7.454, P=0.489 and Q=10.542, P=0.649, respectively). MR-Egger regression revealed no evidence of a pleiotropic influence through genetic variants (intercept -0.004; P=0.101). The leave-one-out sensitivity analysis results suggested that no SNP significantly influenced the link between periodontitis and osteoporosis (Figure 4).

**Table 2:** Association of periodontitis on osteoporosis using various methods.

Method	Beta	SE	OR	95%Cl	P value
IVW (random effects)	0.031	0.056	1.032	0.923-1.153	0.574
IVW (fixed effects)	0.031	0.058	1.032	0.920-1.158	0.588
Simple mode	0.030	0.141	1.031	0.780-1.361	0.835
Weighted mode	0.113	0.086	1.120	0.944-1.328	0.229
Simple median	0.003	0.090	1.003	0.839-1.197	0.977
Weighted median	0.075	0.081	1.078	0.921-1.262	0.346
Penalized weighted median	0.075	0.081	1.078	0.919-1.264	0.351
MR-Egger	0.307	0.157	1.360	0.998-1.853	0.092

IVW: inverse variance-weighted; MR: Mendelian randomization.

## Discussion

This two-sample MR study is the first to examine the causal relationship between genetically predicted periodontitis and osteoporosis. Our findings did not support the impact of genetic periodontitis on osteoporosis risk.

Although bone resorption is a feature of both osteoporosis and periodontitis, the causes of bone resorption in the two diseases are different. Osteoporosis results from degenerative systemic bone loss, leading to skeletal cancellous microstructure loss and fractures. Periodontitis bone loss is the result of local inflammation following an infectious breach of the alveolar cortical bone leading to tooth loss (18). Numerous observational investigations have provided convincing evidence for the link between osteoporosis and periodontitis. For example, the most extensive observational study on periodontitis and osteoporosis was completed by Hong et al. (10). They performed a cross-sectional study of epidemiological data from the KoGES between 2004 and 2016. The study included 125,324 participants aged 40-79 years, and found an adjusted odds ratio (aOR) of periodontitis for osteoporosis of 2.16 (95%CI: 2.01-2.31; P<0.001) and an aOR of periodontitis for any fracture of 1.54 (95%CI: 1.46-1.62; P<0.001). This result indicates an impact of periodontitis on osteoporosis or fractures. The authors suggested regular oral hygiene and checking bone mineral density to prevent periodontitis and osteoporosis from worsening (10). A population-based cohort study in Taiwan enrolled 29,463 individuals with recently discovered periodontitis that were followed up for six years from 2002 to 2008. In the periodontitis cohort and comparison group, incidence rates of osteoporosis were 2.72 and 1.66 per 1000 person-years, respectively. According to a log-rank analysis, accumulated osteoporosis incidence was notably higher in patients with periodontitis than in the control group (P<0.0001) (11). In the most recent meta-analysis, cross-sectional studies (OR=2.17, 95%CI: 1.80-2.61), case-control studies (OR=2.63; 95%CI: 1.69-4.09), and cohort studies (OR=1.70, 95%CI: 1.16-2.49) revealed an increased risk of osteoporosis in patients with periodontitis (19).

However, the exact relationship between periodontitis and osteoporosis has not yet been determined. This relationship seems to be explained by biology. As a result of periodontal disease, pathogenic bacteria may move from the periodontal pocket to the blood, causing the release of interleukin (IL)-6, which probably starts a chain of events resulting in systemic bone resorption (20). Recently, an intriguing bioinformatics study attempted to establish an internal relationship between periodontitis and osteoporosis. The authors discovered a shared mechanism between periodontitis and osteoporosis via genes related to crosstalk and pyroptosis (21). The essential genes *PRKCB*, *GSDMD*, *ARMCX3*, and *CASP3* affected periodontitis and osteoporosis by involving the MAPK signaling pathway and the neutrophil extracellular trap formation (21). Research is still needed for a better understanding of the internal molecular mechanisms of osteoporosis and periodontitis.

Currently, most of the evidence comes from observational cohort studies, which are subjected to confounding factors, leading to biased results. Confounding factors must be adjusted for in the investigation of causal relationships (22). Unfortunately, it is not easy to eliminate confounding. Moreover, positive results are always quickly published, but potential biases must not be ignored (23).

Several factors can influence the validity of the results. First, osteoporosis is a systemic bone disease. This disease induces loss of alveolar bone density, affecting the tooth sockets (24). However, there are significant individual differences in the alveolar bone's structure and integrity that must be considered. Second, osteoporosis drugs, such as estrogen replacement therapy and bisphosphonates, are increasingly prescribed for periodontitis (25). Treatment-related variables may also produce bias, and future observational studies should consider the influence of drug treatment. Third, differences in diagnostic procedures and outcome interpretations can affect results. For instance, radiological criteria for diagnosing periodontitis are discrete. This method usually indicates a positive correlation between periodontitis and osteoporosis. However, the definition of periodontitis by clinical manifestation varies. Studies using this diagnosis approach can reach different conclusions regarding the association between periodontitis and osteoporosis (12). In this study, we used instrumental variables related to diagnosed periodontitis. In previous observational studies, the outcomes are not only periodontitis but also other dental clinical features, such as periodontal symptoms, plaque/gingival bleeding index, sulcus alteration, and missing teeth. Therefore, definitive treatment interventions and specific cause-and-effect relationships cannot be established at this time.

Our study has some limitations that must be considered. First, since this study included both European and Asian populations, the results of this study should be taken with caution when generalized to other ethnic groups. Second, evidence has indicated that subjects in the UK Biobank have poor representativeness because of low participation and healthy volunteer bias. Therefore, further studies are warranted to evaluate to what degree our findings may be generalized to the general population (26). Third, gender, which is a relevant aspect of susceptibility, age, smoking habits, and general health are important aspects related to the development of periodontitis. However, these factors were not fully adjusted for in the GWAS data, so they may somewhat affect the final results. However, our results are negative. Therefore, it is unlikely that the non-adjustment of these factors affected our final conclusion.

### Conclusion

Our results did not show a significant detrimental effect of periodontitis on the risk of osteoporosis.

## Supplementary Material

Click here to view [pdf].
