# Supplementary material for: Periodontitis and osteoporosis: a two-sample Mendelian randomization analysis
Source: Braz J Med Biol Res. 2024 Mar 18;57:e12951. doi: 10.1590/1414-431X2024e12951 (PMC10946243; doi:10.1590/1414-431X2024e12951)
Supplement: Supplementary file 1 [file 1414-431X-bjmbr-57-e12951-suppl.pdf]

Table S1. Description of data sources.

Traits	type	Data sources (ID)	Sample size	Cases	Controls	Gender	Access link
Periodontitis	Exposure	Gene-Lifestyle Interaction in the Dental Endpoints (GLIDE) Consortium	45,637	17,353	28,284	Males and Females	https://www.nature.com/articles/s41467-019-10630-1
Osteoporosis	Outcome	UKBiobank (bbj-a-137)	212,453	7,788	204,665	Males and Females	https://gwas.mrcieu.ac.uk/datasets/bbj-a-137/
